# Development of a prognostic model based on ferroptosis-related genes for colorectal cancer patients and exploration of the biological functions of NOS2 *in vivo* and *in vitro*

**DOI:** 10.3389/fonc.2023.1133946

**Published:** 2023-06-06

**Authors:** Hongming Li, Xiaochuang Feng, Yong Hu, Junjiang Wang, Chengzhi Huang, Xueqing Yao

**Affiliations:** ^1^ The Second School of Clinical Medicine, Southern Medical University, Guangzhou, Guangdong, China; ^2^ Department of Gastrointestinal Surgery, Department of General Surgery, Guangdong Provincial People’s Hospital (Guangdong Academy of Medical Sciences), Southern Medical University, Guangzhou, China; ^3^ Department of Colorectal Surgery, Guangdong Provincial Hospital of Chinese Medicine, Guangzhou, Guangdong, China; ^4^ School of Bioscience and Bioengineering, South China University of Technology, Guangzhou, Guangdong, China

**Keywords:** colorectal cancer, ferroptosis, prognostic model, gene, NOS2

## Abstract

**Background:**

Ferroptosis is involved in many malignant tumors and has been implicated in important mechanisms of colorectal cancer (CRC) suppression. However, the prognostic and predictive values of the ferroptosis activation pattern in CRC patients have not been noted. Here, we aimed to construct and validate a prediction model based on ferroptosis-related genes (FRGs) for CRC patients and investigated the expression pattern and biological function of the most significantly altered gene.

**Methods:**

A total of 112 FRGs were obtained from the FerrDb website, and the clinical characteristics of 545 CRC patients and their global gene expression profiles were downloaded from The Cancer Genome Atlas (TCGA) database. Survival-related FRGs were identified by Cox proportional hazards regression analysis. Finally, the expression pattern and biological function of NOS2, the most implicated gene was explored *in vitro* and *in vivo*.

**Results:**

The prediction model was established based on 8 FRGs. Patients in the high- or low-risk group were stratified based on the median risk value calculated by our model, and patients in the high-risk group experienced poor overall survival (*p*<0.01). Further validation demonstrated that the FRG model acted as an independent prognostic indicator for CRC patients (HR=1.428, 95% CI, 1.341-1.627; *p*<0.001). The area under the receiver operating characteristic (ROC) curve (AUC) for 5-year survival was 0.741. NOS2 was one of the most significantly affected FRGs and was highly expressed in malignant tissue, but it inhibited tumor growth and induced tumor cell death *in vitro* and *in vivo*, possibly by repressing the NF-κB pathway.

**Conclusion:**

Our study revealed that FRGs have potential prognostic value in CRC patients and that NOS2 suppresses tumor progression, providing a novel therapeutic target for CRC treatment based on ferroptosis.

## Introduction

CRC is the third most frequently diagnosed malignant tumor worldwide, accounting for approximately 10% of all cancers and leading to almost 9×10^5^ deaths annually (1, 2). The incidence of CRC has been increasing over recent decades, and it is foreseeable that new cases will reach 2.5 million in 2035, and the treatment and management of CRC have become more difficult due to the increase in drug resistance (3–5). However, guiding prognostication and treatment decision-making with biomarkers would provide promising therapeutic targets for CRC (6).

Ferroptosis is an important and recently identified form of nonapoptotic cell death driven by iron-dependent lipid peroxidation that was first proposed in 2012 (7). Emerging evidence has gradually indicated the tumor-suppressive consequence of ferroptosis through cysteine deprivation and reactive oxygen species (ROS) production by p53 (8, 9). To date, ferroptosis has been shown to affect the immune microenvironment, metabolism, and cell proliferation in CRC and acts downstream of chemotherapy and targeted therapy in KRAS-mutated CRC cells (10–12). However, the diagnostic and prognostic values and underlying biological mechanisms involved in ferroptosis remain unclear in CRC.

As shown in previous studies, NOS2 (Enzyme Nitric Oxide Synthase 2) might act in the process of ferroptosis and have implications for patient stratification for prognosis (13, 14). NOS2 is a calcium-independent and inducible enzyme that contributes to the production of NO in cells; therefore, it is related to immune response facilitation, the vascular relaxation function, and inflammation (15, 16). In many types of cancer, the mechanisms by which NOS2 is involved are complex and poorly defined, with both promoting and inhibiting functions having been described (15, 17, 18). Several studies have addressed the mechanisms by which NOS2 promotes tumor progression by p53 and TNFα interactions within the tumor microenvironment (19, 20). However, NOS2 is essential for T cell immunotherapies to destroy tumors (21).

Our study analyzed the correlation between the expression pattern of FRGs and the survival of 545 CRC patients from the TCGA database, and a prognostic model based on the risk score of 8 FRGs identified by multivariate Cox regression analysis was established. Furthermore, we explored the expression pattern and the tumor suppressive role and mechanism of NOS2, which was one of the most significantly affected gene in our model.

## Materials and methods

### Data sources

A total of 112 FRGs, including ferroptosis drivers, suppressors and markers, were obtained from the FerrDB website (http://www.zhounan.org/ferrdb/). We downloaded the mRNA expression data and clinical characteristics of 545 patients diagnosed with CRC from The Cancer Genome Atlas (TCGA) database (https://www.cancer.gov/).

### Identification of differentially expressed FRGs and enrichment analysis

We ran the edgeR package to identify differentially expressed FRGs (fold change >2, adjusted *p*-value< 0.05) between CRC and normal tissues. Then, we used a parallel box diagram to visualize these eligible FRGs and conducted Gene Ontology (GO) and Kyoto Encyclopedia of Genes and Genomes (KEGG) analyses for gene functional enrichment analyses. “GO plot” and “KEGG plot” were used to visualize the results.

### Establishment of the individualized prognostic model based on FRGs

We performed univariate Cox regression analysis to select the significant survival-related FRGs and avoid false positives and overfitting of the model by LASSO regression analysis. Next, we used multivariate Cox regression analysis to further identify FRGs that could independently predict survival. Finally, the prognostic model was established according to the relative expression levels of the screened FRGs and weighted according to regression coefficients (β) with a multivariate Cox regression model. The equation was as follows:


$$\begin{array}{c} \text{Risk score }=\;\beta \text{gene }\left(1\right)\times \text{FRGexpression }\left(1\right)\;+\text{ βgene }\left(2\right)\;\times \text{FRGexpression }\left(2\right)\;+\cdot \cdot \cdot \\ +\beta \text{gene }\left(n\right)\;\times \text{FRGexpression }\left(n\right) \end{array}$$

### Calculating survival and the risk score

According to the median risk score, CRC patients were divided into the high- or low-risk group. Kaplan-Meier curves were generated to analyze the overall survival (OS) times between the two groups, and a time-dependent receiver operating characteristic (ROC) curve was used to evaluate the accuracy of the prediction model. Then, we drew a nomogram to demonstrate the predictive probability and observation rate of five-year OS in CRC patients.

### Western blot analysis and quantitative real-time PCR

We used RIPA buffer (Amresco, America) to lyse cells and tissues, separated total proteins by 10% SDS-PAGE (Amresco, America) and transferred them to PVDF membranes. All membranes were incubated overnight with primary antibodies at 4°C and with the HRP-conjugated secondary antibody at room temperature for 1 h. All western blot results were quantified by software Image J (v1.8.0).

For qRT-PCR, total mRNA was extracted from tumor cells or tumor tissues using TRIzol reagent and reverse transcribed into cDNA with a PrimeScript RT-PCR Kit. cDNA was amplified using SYBR™ Premix Ex Taq™ (TaKaRa, Japan) on a LightCycler 96 Detection System (Roche). GAPDH CT values were used for normalization.

### MTT proliferation and clonogenic assays

For the MTT (3-(4, 5-dimethyl-thiahiazo-2-yl)-2, 5-di- phenytetrazolium bromide) proliferation assay, transfected cells (1×10^3^ cells per well) were seeded onto 96-well plates. After 24 h, we performed an MTT assay at fixed time points every day. For the clonogenic assay, 500 cells were cultivated per well into 6-well plates and maintained in RPMI 1640 medium with 10% fetal bovine serum at 37°C for 7 days.

### *In vivo* subcutaneous xenograft models

All nude mice were purchased from Guangdong Medical Laboratory Animal Center. NOS2-overexpressing and control cell lines were transplanted subcutaneously into the bilateral flanks, and appropriate care was given to these animals. Tumor volume [(length × width^2^)/2] was measured every 3 days, and all mice were sacrificed 21 days after injection.

### Statistical analysis

All statistical analyses were performed using GraphPad Prism 8.0 and R 3.6.2. The R package edgeR was used for differential expression analysis, and then univariate LASSO and multivariate Cox regression analyses were performed to identify FRGs associated with prognosis and further introduced into the prognostic model. Differences in OS between CRC patients in the high-risk group and low-risk group were generated with the Kaplan-Meier method. The R package “survivalROC” was run to generate the ROC curve and the corresponding area under the ROC curve (AUC) for model evaluation. Relevant R packages used for statistical analysis referenced the method in (22).

Data are shown as the mean ± SD, and all tests were considered statistically significant only when *p*< 0.05 was achieved.

## Results

### Differentially expressed FRGs in CRC and functional enrichment

**Figure 1:**
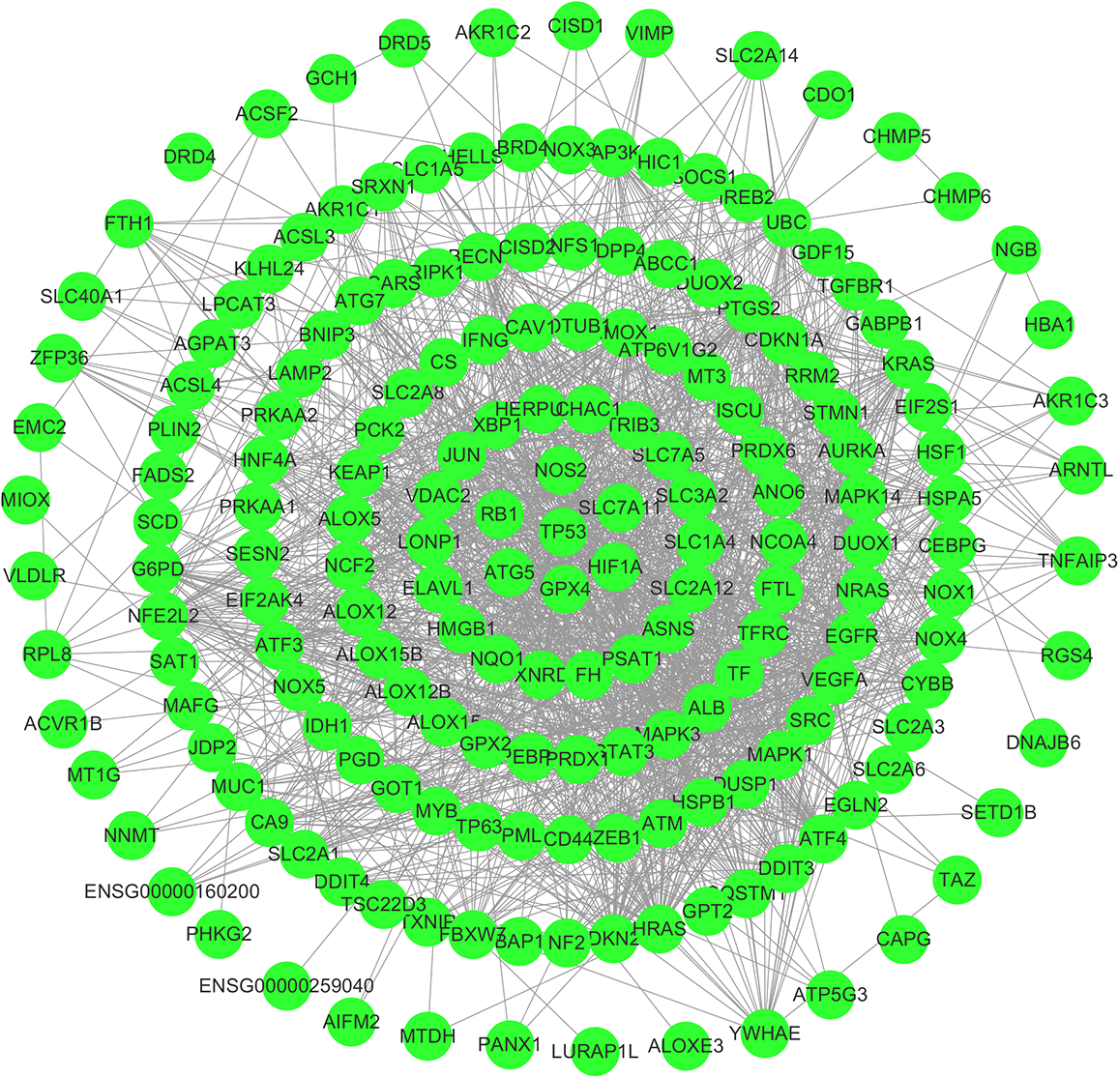
112 FRGs were obtained from the FerrDB website, and the interaction network was constructed by cyberscope software.

**Figure 2:**
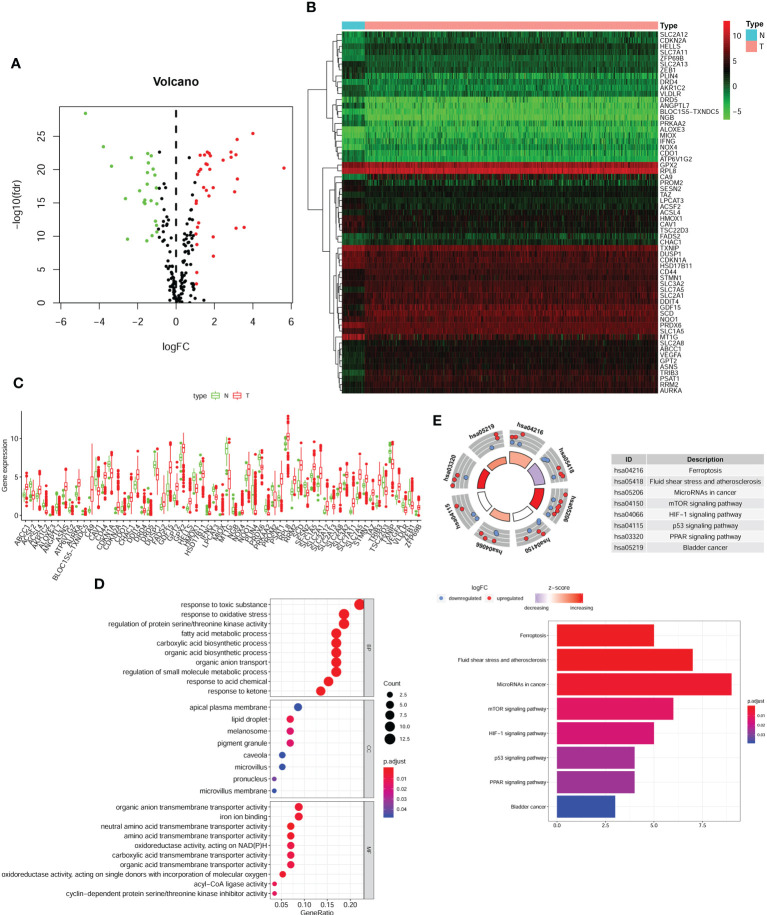
Differentially FRGs expressed in CRC and functional enrichment. Volcano plot **(A)**, heatmap **(B)** and expression bar chart **(C)** showing the 61 differentially expressed FRGs in CRC tissues compared with normal tissue. The red dots represented significantly up-regulated FRGs, green dots standed for FRGs with significantly downregulated and black dots standed for no significant differences FRGs. The GO analysis **(D)** and KGEE analysis **(E)** for molecular functions and potential pathways for differentially expressed FRGs involved in.

First, we downloaded RNA-seq and clinical data from 646 CRC tissue samples and 68 normal colorectal mucosa specimens (Paired colon samples were from partial colon resection for carcinoma) from the TCGA database. Altogether, 545 CRC patients with follow-up data were eligible (**Table 1**). A total of 112 FRGs were accessed from the FerrDB website (**Figure 1**), and 61 genes (40 upregulated and 21 downregulated) were obtained under the criteria FDR<0.05 and log2 (fold change) > 1 (**Figures 2A, B**). Box plot graph showing these differential genes expression between normal and tumor tissues (**Figure 2C**). These differentially expressed FRGs were then subjected to functional enrichment analysis, and the top 28 GO terms and 8 KEGG pathways are visualized in **Figures 2D, E**. The top ranked pathways according to enrichment score were “Ferroptosis” and “Response to toxic substance”.

**Table 1:** Specific baseline clinical characteristic of 545 colorectal cancer patients.

Characteristic	
Gender	
Male	291
Female	254
Age	
<60	153
≧60	392
Stage	
I/II	305
III/IV	225
unknow	15
Pathologic T stage	
T1-2	111
T3-4	433
Unknow	1
Pathologic N stage	
N0	322
N1-2	222
unknow	1
Pathologic M stage	
M0	406
M1	76
unknow	63
Survival time	
0-3 years	441
3-5 years	67
>5 years	37

### Identification of prognostic FRGs and construction of a predictive model

**Figure 3:**
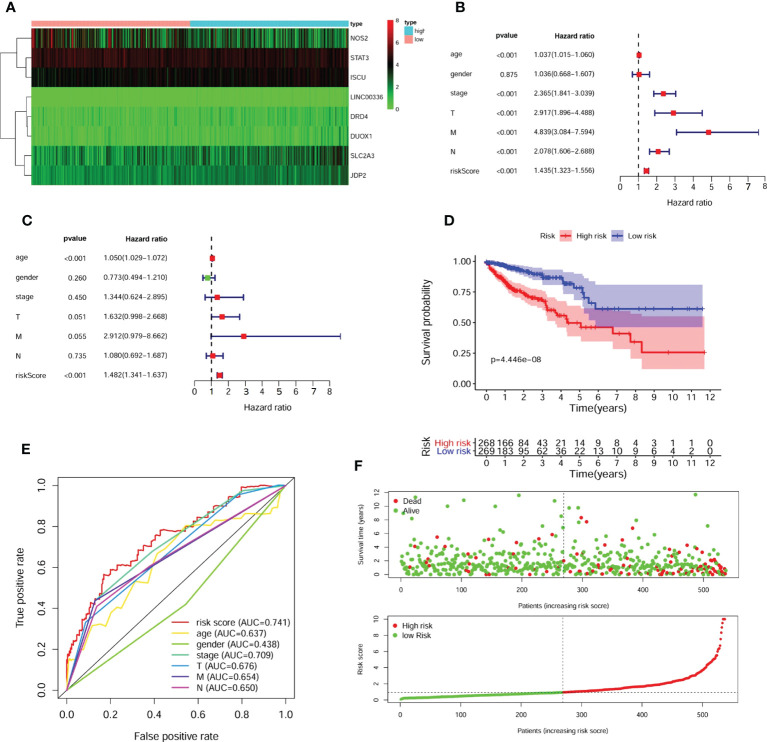
Identification of prognostic FRGs and construction of predictive model. **(A)** The heatmap of 8 included FGRs expression profile. A forest plot of univariate **(B)** and multivariate **(C)** Cox regression analysis for CRC patients. The K-M survival curve **(D)**, distribution of prognostic index and survival status **(E)** of 545 CRC patients in low- and high-risk groups. **(F)** The ROC curves validated the prognostic significance of risk score based on FRGs and other clinical indicators.

We selected the above 61 FRGs for further exploration and performed univariate Cox regression analysis. The results revealed that 14 differentially expressed FRGs were significantly correlated with OS (**Table 2**). Then, we conducted LASSO regression analysis to narrow the scope and avoid false positives and ultimately identified 8 FRGs independently associated with survival in CRC patients by multivariate Cox regression to construct a predictive model (**Supplementary Table 1**). A heatmap of the expression profiles of 8 FRGs is shown in **Figure 3A**.

**Table 2:** Fourteen prognosis-related genes obtained based on univariate COX regression analysis.

Gene symbol	Hazard ratio	95%CI	*p-*Value
**HSPB1**	1.228	1.002−1.504	0.048
**DDIT3**	1.314	1.022−1.690	0.033
**NOS2**	0.852	v0.747−0.973	0.018
**DRD4**	1.719	1.162−2.542	0.007
**STAT3**	0.616	0.390−0.975	0.039
**LINC00336**	4.285	1.352−13.578	0.013
**NOX4**	1.612	1.015−2.561	0.043
**ATP6V1G2**	6.506	1.986−21.315	0.002
**SLC2A3**	1.348	1.102−1.649	0.004
**JDP2**	2.214	1.386−3.539	<0.001
**DUOX1**	1.800	1.177−2.752	0.007
**SLC2A6**	1.459	1.061−2.006	0.020
**ISCU**	1.865	1.043−3.334	0.036
**ALOX12**	2.561	1.196−5.482	0.015

According to the model, 545 patients were classified into the high- or low-risk group based on the median risk scores [Risk score = -0.144 ×NOS2 expression + 0.723 × DRD4 expression + (-0.561) × STAT3 expression + 1.208 × LINC0036 expression + 0.431 × SLC2A3 expression + 0.707 × JDP2 expression + 0.770 × DUOX1 expression+ 0.996 × ISCU expression]. Subsequently, uni- and multivariate Cox regression analyses demonstrated that the risk score acted as an independent risk factor and an independent prognostic factor for the survival of CRC patients (**Figures 3B, C**). A K-M survival curve indicated that the survival rate of CRC patients in the high-risk group was significantly lower than that of CRC patients in the low-risk group (**Figure 3D**). The survival statuses of CRC patients in the two groups were presented in **Figure 3E**. The ROC curve for 5-year survival prediction and AUC for the risk score model showed good accuracy, and the area under the ROC curve was 0.741, which was higher than that of the ROC curve for age (0.637), sex (0.438), disease stage (0.709), T stage (0.676), N stage (0.654) and M stage (0.650) (**Figure 3F**).

### Evaluation of the accuracy of the predictive model

**Figure 4:**
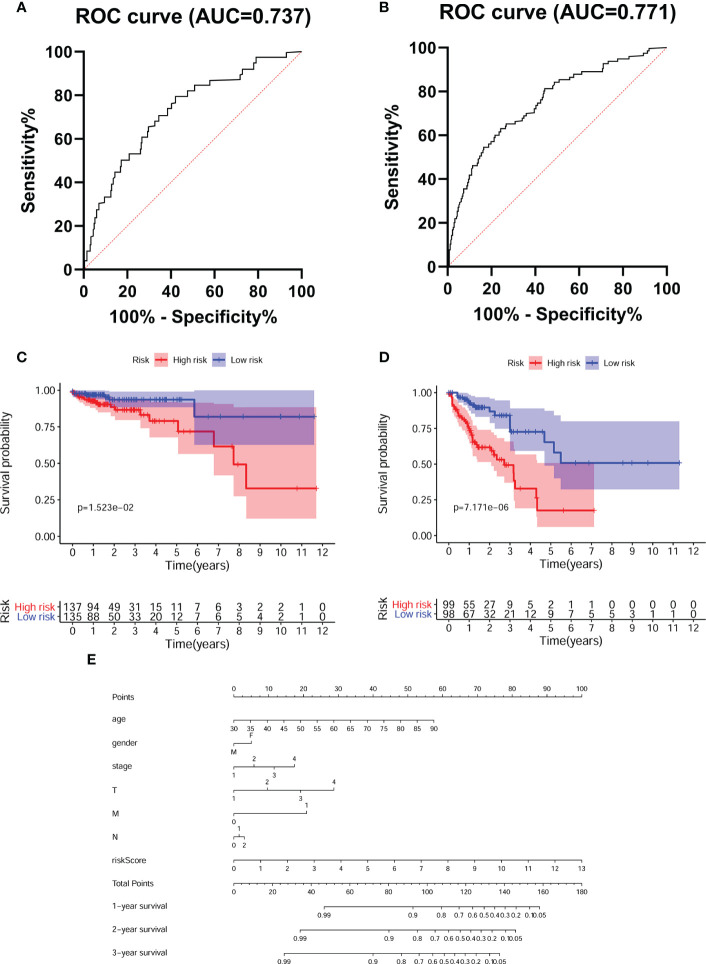
Evaluated the accuracy of the predictive model. Tumor stage-dependent ROC curve analysis **(A)**.I/II; **(B)** III/IV) for survival prediction based on the model. K-M survival curves for CRC patients with different tumor stage **(C)**.I/II; **(D)** III/IV) in low- and high- risk groups. **(E)** Nomogram predicts the probability of 3 years overall survival (OS) in CRC patients.

To assess the prognostic efficacy of our model, we performed disease stage-based ROC curve analysis. The stage I/II and III/IV AUC values of the predictive model were 0.737 and 0.771, respectively, reflecting the superior performance of the FRG model for CRC prognostication (**Figures 4A, B**). Moreover, the K-M survival curve showed that the survival rates of CRC patients with stage I/II and III/IV disease in the high-risk group were distinctly lower than those in the low-risk group (**Figures 4C, D**). Finally, we constructed a nomogram to predict 1-year, 2-year and 3-year survival according to age, sex, clinical stage and our predictive model (**Figure 4E**).

### NOS2 might act as a protective factor

**Figure 5:**
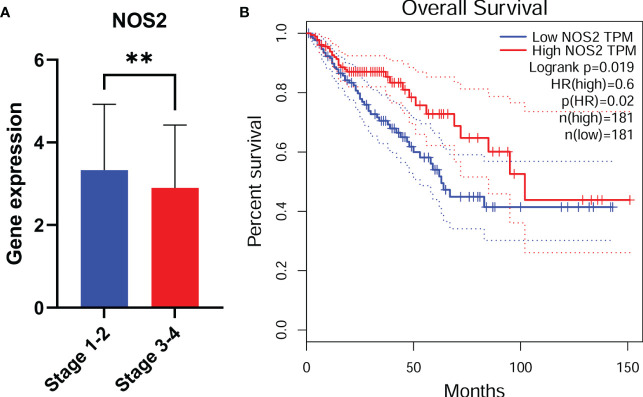
NOS2 might act as a protective factor. NOS2 expression among different tumor stage **(A)** in CRC. **(B)** CRC patients with low NOS2 expression had a shorter overall survival. **, p<0.01.

Considering that NOS2 was one of the most significantly affected FRGs in our model (**Supplementary Table 1**) and the most obvious expression differences between patients in high risk and low risk groups (**Figure 3A**), we further investigated the role of NOS2 in ferroptosis-related tumor progression. The expression of NOS2 in 545 CRC patients with early or advanced TNM stages was detected. Overall, the expression of NOS2 gradually decreased as the TNM stage advanced (**Figure 5A**). Furthermore, the survival rates of CRC patients with high NOS2 expression were significantly higher than those of patients with low NOS2 expression (**Figure 5B**). Taken together, these data suggest that NOS2 might have a tumor suppressive function in CRC.

### NOS2 suppresses tumor proliferation *in vitro*

**Figure 6:**
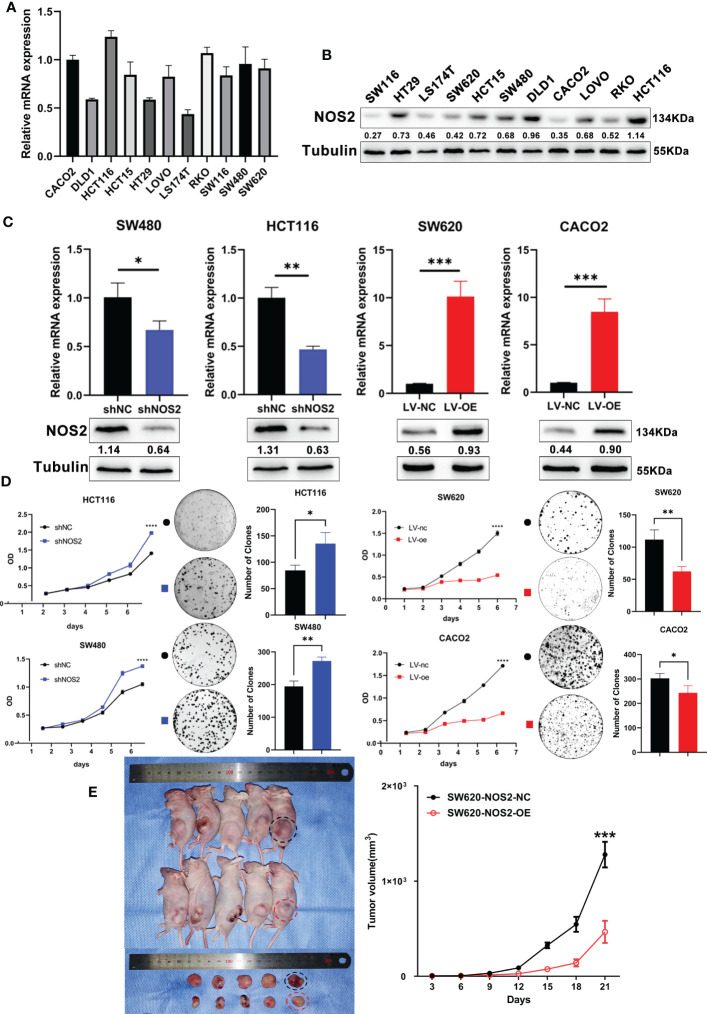
NOS2 suppresses tumor proliferation *in vitro* and vivo. **(A)** q-PCR and **(B)** western blot determined the endogenous expression of NOS2 in 11 CRC cell lines. **(C)** q-PCR and western blot results of NOS2 expression upon knockdown and overexpression of NOS2 in different cell lines. **(D)** MTT assay (Left) and clonogenic assay (Right) on cell proliferation ability. **(E)** Subcutaneous xenograft tumor model and growth rates of tumor xenografts inoculated subcutaneously. p>0.05; *, p<0.05; **, p<0.01; ***, p<0.001.

To further explore the biological functions of NOS2 in CRC, we first detected endogenous NOS2 expression in 11 CRC cell lines through qRT-PCR and western blot (**Figures 6A, B**). According to the results, NOS2 was relatively highly expressed in HCT116 and SW480 cells and weakly expressed in SW620 and CACO2 cells. Thus, we generated HCT116 and SW480 cell lines stably overexpressing NOS2 and SW620 and CACO2 cell lines with NOS2 knockdown. The lentiviral transfection efficiency of overexpression and knockdown was determined by qRT-PCR and western blot (**Figure 6C**).

Then, several experiments were performed to determine whether NOS2 affects the biological functions of CRC cells. The results of the MTT assay indicated that elevated NOS2 reduced cell proliferation, while the growth rate of cell lines increased when NOS2 was knocked down (**Figure 6D**).

To assess the function of NOS2 with respect to tumorigenic inhibition *in vivo*, SW620 NOS2 overexpression and control cell lines were used in subcutaneous tumorigenesis assays. The result demonstrated that elevated NOS2 expression decreased tumorigenicity in nude mice (**Figure 6E**). Together, these results suggest that NOS2 mainly functions as a tumor suppressor in CRC.

### NOS2 inhibits the NF-κB signaling pathway

**Figure 7:**
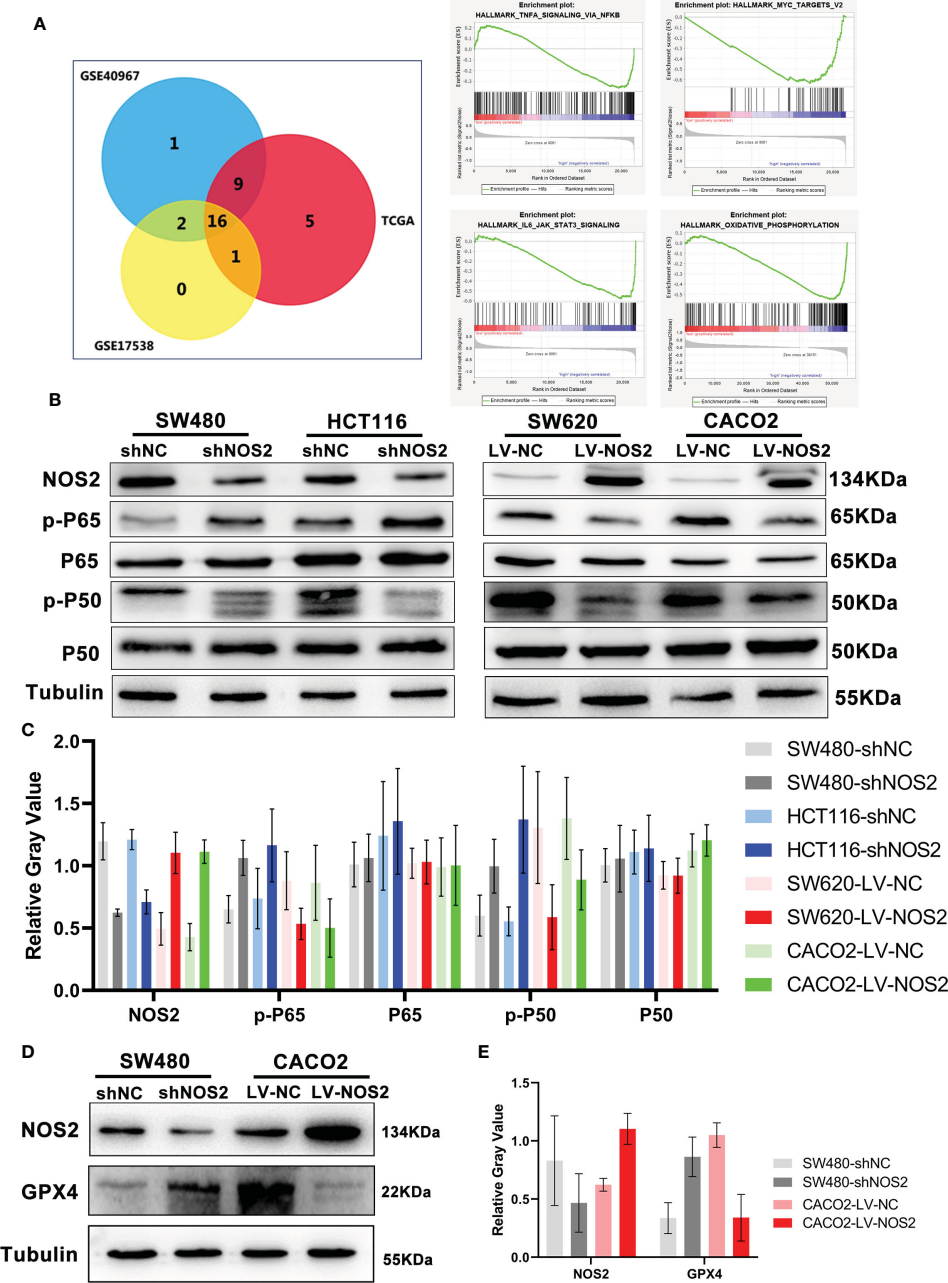
NOS2 inhibited NF-κB signaling pathway. **(A)** The wayne figures of overlapping and different enrichment pathways between different NOS2 expression samples in 3 databases. The NF-κB signaling pathway was the most significantly enriched pathway. **(B)** NF-κB activation in different cell lines with NOS2 knockdown and overexpression was monitored by western blot analysis. **(C)** Relative expression of NOS2 and NF-κB signaling proteins. The data were expressed as the ratio of specific protein level (gray value) to Tubulin protein level (gray value). **(D)** Western blot results of GPX4 expression upon knockdown and overexpression of NOS2 in CRC cells. **(E)** Relative expression of NOS2 and GPX4.

To further demonstrate the downstream molecular mechanism of NOS2, we used GSEA software to explore the related signaling pathways in microarray data from the TCGA, GSE17538 and GSE40967. The “NF-kappa B signaling pathway”, “IL6-STAT3 signaling pathway”, “c-MYC signaling pathway” and “oxidative phosphorylation” were highly enriched and associated with NOS2 knockdown (**Figure 7A**).

We initially detected the relationship between NOS2 and STAT3, c-MYC pathways. Overall, NOS2 expression does not affect p-STAT3 and c-MYC changes (**Supplementary Figure 1**). Therefore we explored the association of NOS2 with NF-κB pathway.

Moreover, the western blot results suggested that NOS2 knockdown in SW480 and HCT116 cells increased the expression of p-P50 and p-P65, whereas NOS2 overexpression in SW620 and CACO2 cells reduced the expression of p-P50 and p-P65 (**Figures 7B, C**). Finally, to verify the regulatory relationship between NOS2 and ferroptosis, we conduct western blot and the outcome showed a higher level of GPX4 when NOS2 was knockdown, whereas NOS2 overexpression in CACO2 cells decreased the expression of GPX4 (**Figures 7D, E**).

In conclusion, NOS2 might inhibit CRC carcinogenicity *via* suppression of the NF-κB signaling pathway.

## Discussion

Ferroptosis is a recently discovered type of nonapoptotic mechanism involved in excessive lipid peroxidation and iron-dependent damage to membrane lipids (23, 24). Numerous studies have shown that the peroxidation of phospholipids (PLs), especially arachidonic acid, is mainly responsible for ferroptosis induction, while cumulative GPX4 and the inactivation of ACSL4 can attenuate ferroptosis by reducing lipid alcohol conversion and PL biosynthesis, respectively (24–26). At the organoid level, significant changes in mitochondrial morphology usually lead to increased membrane density, condensation or swelling and rupture of the outer membrane (27, 28).

Accumulating studies have suggested that ferroptosis participates in human diseases through a variety of mechanisms, the most likely of which is tumor suppression (23). The underlying mechanism of tumor suppression through ferroptosis in CRC remains to be investigated. In KRAS-mutant CRC cells, combination treatment with β-elemene and cetuximab enhanced the cytotoxic effect against cancer cells by inducing ferroptosis and inhibiting EMT (29). Moreover, it has been reported that the compound IMCA can upregulate SLC7A11, resulting in ROS accumulation and promoting ferroptosis (30). GPX4 is the core marker of ferroptosis, which protects cells from oxidative stress, and degradation of GPX4 contributes to ferroptosis (31). In our research, GPX4 expression was negatively correlated with the survival of CRC patients, which demonstrated the tumor inhibition effect of ferroptosis in CRC (**Supplementary Figure 2**). Thus, ferroptosis-inducing agents might be a potential therapeutic option for CRC treatment (32).

The exploration of ferroptosis and FRGs aimed to develop effective biomarkers for CRC prognosis prediction and therapy monitoring. In this study, we identified 8 candidates FRGs from 214 FRGs according to the FerrDb website and TCGA database and constructed a CRC predictive model. Further calculations revealed a high correlation between the survival outcomes of CRC patients and the risk score, as confirmed by uni- and multivariate Cox regression analyses. The survival rate of CRC patients in the high-risk group was significantly lower than that of CRC patients in the low-risk group, and the ROC curve for 5-year survival prediction and AUC for the risk score model showed good accuracy. These results revealed that our prognostic model, which was retrospectively validated in CRC patients at risk for mortality, had a good fit and predictive ability.

According to previous studies, the 8 FRGs selected for model construction play an important role by functionally inhibiting or promoting tumor progression in different tumor types. According to our data, NOS2 was one of the most significantly affected FRGs, and the most obvious expression differences between high risk and low risk groups patients, therefore we began to explore the biological functions and the molecular mechanism of NOS2 in CRC. NOS2 is an inducible isoform of NOS enzymes and functions as a key inflammatory enzyme responsible for nitric oxide biosynthesis (33). Recent studies connected NOS2 and ferroptosis were almost based on bioinformatic analysis, and NOS2 had been identified as the marker of ferroptosis functions in the process of HIF-1 signaling pathway, NOD-like receptor signaling pathway, central carbon metabolism and macrophage polarization (13, 34, 35). The dysregulation of NOS2 expression can be observed under pathological conditions, including cytokine exposure, inflammation and tumors (33, 36). A large number of studies have considered NOS2 to be a promoter and a prognostic indicator for malignancy progression. In hepatocellular carcinomas, NOS2 is a Wnt β-catenin/Tcf-4 target gene that promotes tumorigenesis (37). However, NOS2 has also been proven to have both antitumoricidal functions and tumor suppressive properties in various tumors.

It was shown that a high level of NO induced the phosphorylation and stabilization of p53 (38). In patients with ulcerative colitis or Crohn’s disease, NOS2 and p-p53 are colocalized in tissues (36). Moreover, in several trials, selective or nonselective NOS2 inhibitors did not have a therapeutic benefit in some diseases (39–41). Thus, the underlying molecular mechanisms by which NOS2 promotes the progression of CRC have remained complex and need to be further explored. In our model, considering that NOS2 was the most prominent gene and that decreased NOS2 expression was clearly linked to a poor prognosis, we performed biological function experiments *in vitro* and *in vivo*. The results suggested that elevated NOS2 significantly inhibited CRC cell proliferation and promoted apoptosis.

Though NOS2 had been reported involving in the development of tumors in our research, the underlying molecular mechanism is still unclearly elucidated. Our further exploration of the molecular mechanism preliminarily revealed that the inhibition of NF-κB signaling might be an important contributor to CRC when NOS2 is upregulated. In breast cancer, the co-expression of NOS2 and COX2 is involved in the regulation of oncogenic pathways such as ERK, PI3K and NF-κB results in a poor prognosis (42, 43). Among the inflammatory diseases, NOS2 might inhibit the phosphorylation of NF-κB (44). Our results showed that the expression level of NOS2 could induce the opposite expression of GPX4, which might demonstrate that NOS2 can participate in GPX4 synthesis or breakdown, repress NF-κB pathway by inhibiting the phosphorylation of the p50 and p65, and thus regulated the ferroptosis in CRC cells.

Nevertheless, this study was subject to several limitations. First, it was a retrospective study, and selection bias cannot be ruled out. Second, although effective external verification was performed, internal data validation is still lacking. In addition, the molecular mechanisms underlying the 8 identified FRGs need to be further explored.

In summary, our research demonstrated, for the first time to our knowledge, the potential prognostic value of FRGs in CRC patients. The construction of a predictive model based on FRGs may be helpful for decision-making in clinical practice. In addition, our results suggest that NOS2 might inhibit CRC cell growth and induce apoptosis by inhibiting NF-κB signaling pathways *in vitro* and *in vivo*.

## Data availability statement

The original contributions presented in the study are included in the article/**Supplementary Material**, further inquiries can be directed to the corresponding authors.

## Ethics statement

The animal study was reviewed and approved by Ethics Committee of Guangdong Provincial Hospital of Chinese Medicine.

## Author contributions

XY and HL designed the study. XF, YH, and JW performed the molecular biology experiments and statistical analysis. XY, CH, and HL contributed to administrative, technical, or material support. HL and XF wrote the manuscript. All authors contributed to the article and approved the submitted version.

## Supplementary material

Supplementary Figure 1 Different NOS2 expression level hardly affected STAT3 and c-MYC signaling pathway.

Supplementary Figure 2 Kaplan-Meier survival curves of OS with GPX4 expression level.

## References

[1] Dekker E Tanis PJ Vleugels JLA Kasi PM Wallace MB. Colorectal cancer. Lancet (2019) 394(10207):1467–80. doi: 10.1016/S0140-6736(19)32319-0 31631858

[2] Bray F Ferlay J Soerjomataram I Siegel RL Torre LA Jemal A. Global cancer statistics 2018: GLOBOCAN estimates of incidence and mortality worldwide for 36 cancers in 185 countries. CA Cancer J Clin (2018) 68(6):394–424. doi: 10.3322/caac.21492 30207593

[3] Arnold M Sierra MS Laversanne M Soerjomataram I Jemal A Bray F. Global patterns and trends in colorectal cancer incidence and mortality. Gut (2017) 66(4):683–91. doi: 10.1136/gutjnl-2015-310912 26818619

[4] Sharma R. An examination of colorectal cancer burden by socioeconomic status: evidence from GLOBOCAN 2018. EPMA J (2020) 11(1):95–117. doi: 10.1007/s13167-019-00185-y 32140188 PMC7028897

[5] Duffy MJ Crown J. Drugging “undruggable” genes for cancer treatment: are we making progress? Int J Cancer (2020) 148(1):8–17. doi: 10.1002/ijc.33197 32638380

[6] Sveen A Kopetz S Lothe RA. Biomarker-guided therapy for colorectal cancer: strength in complexity. Nat Rev Clin Oncol (2020) 17(1):11–32. doi: 10.1038/s41571-019-0241-1 31289352 PMC7577509

[7] Dixon SJ Lemberg KM Lamprecht MR Skouta R Zaitsev EM Gleason CE. Ferroptosis: an iron-dependent form of nonapoptotic cell death. Cell (2012) 149(5):1060–72. doi: 10.1016/j.cell.2012.03.042 PMC3367386 22632970

[8] Liang C Zhang X Yang M Dong X. Recent progress in ferroptosis inducers for cancer therapy. Adv Mater (2019) 31(51):e1904197. doi: 10.1002/adma.201904197 31595562

[9] Xie Y Zhu S Song X Sun X Fan Y Liu J. The tumor suppressor p53 limits ferroptosis by blocking DPP4 activity. Cell Rep (2017) 20(7):1692–704. doi: 10.1016/j.celrep.2017.07.055 28813679

[10] Chen P Li X Zhang R Liu S Xiang Y Zhang M. Combinative treatment of beta-elemene and cetuximab is sensitive to KRAS mutant colorectal cancer cells by inducing ferroptosis and inhibiting epithelial-mesenchymal transformation. Theranostics (2020) 10(11):5107–19. doi: 10.7150/thno.44705 PMC7163451 32308771

[11] Zhang Y Song J Zhao Z Yang M Chen M Liu C. Single-cell transcriptome analysis reveals tumor immune microenvironment heterogenicity and granulocytes enrichment in colorectal cancer liver metastases. Cancer Lett (2020) 470:84–94. doi: 10.1016/j.canlet.2019.10.016 31610266

[12] Chapkin RS Navarro SL Hullar MAJ Lampe JW. Diet and gut microbes act coordinately to enhance programmed cell death and reduce colorectal cancer risk. Dig Dis Sci (2020) 65(3):840–51. doi: 10.1007/s10620-020-06106-8 PMC7605510 32006211

[13] He J Li X Yu M. Bioinformatics analysis identifies potential ferroptosis key genes in the pathogenesis of pulmonary fibrosis. Front Genet (2021) 12:788417. doi: 10.3389/fgene.2021.788417 35069688 PMC8770739

[14] Chen Y Li H. Prognostic and predictive models for left- and right- colorectal cancer patients: a bioinformatics analysis based on ferroptosis-related genes. Front Oncol (2022) 12:833834. doi: 10.3389/fonc.2022.833834 35265525 PMC8899601

[15] Vannini F Kashfi K Nath N. The dual role of iNOS in cancer. Redox Biol (2015) 6:334–43. doi: 10.1016/j.redox.2015.08.009 PMC4565017 26335399

[16] Somasundaram V Gilmore AC Basudhar D Palmieri EM Scheiblin DA Heinz WF. Inducible nitric oxide synthase-derived extracellular nitric oxide flux regulates proinflammatory responses at the single cell level. Redox Biol (2020) 28: 2213-17. doi: 10.1016/j.redox.2019.101354 PMC6920088 31683257

[17] Perwez Hussain S. Deciphering the complex biological interactions of nitric oxide in cancer. Redox Biol (2015) 5:413. doi: 10.1016/j.redox.2015.09.011 28162268

[18] Thomas DD Wink DA. NOS2 as an emergent player in progression of cancer. Antioxid Redox Signal (2017) 26(17):963–5. doi: 10.1089/ars.2016.6835 28506076

[19] Fransen K Elander N Soderkvist P. Nitric oxide synthase 2 (NOS2) promoter polymorphisms in colorectal cancer. Cancer Lett (2005) 225(1):99–103. doi: 10.1016/j.canlet.2005.02.006 15922861

[20] Speckmann B Pinto A Winter M Förster I Sies H Steinbrenner H. Proinflammatory cytokines down-regulate intestinal selenoprotein P biosynthesis via NOS2 induction. Free Radic Biol Med. (2010) 49(5):777–85. doi: 10.1016/j.freeradbiomed.2010.05.035 20542496

[21] Marigo I Zilio S Desantis G Mlecnik B Agnellini AH Ugel S. T Cell cancer therapy requires CD40-CD40L activation of tumor necrosis factor and inducible nitric-Oxide-Synthase-Producing dendritic cells. Cancer Cell (2016) 30(4):651. doi: 10.1016/j.ccell.2016.09.009 27728809 10.1016/j.ccell.2016.09.009

[22] Zhu Y Wang R Chen W Chen Q Zhou J. Construction of a prognosis-predicting model based on autophagy-related genes for hepatocellular carcinoma (HCC) patients. Aging (Albany NY). (2020) 12(14):14582–92. doi: 10.18632/aging.103507 PMC7425489 32681721

[23] Stockwell BR Jiang X Gu W. Emerging mechanisms and disease relevance of ferroptosis. Trends Cell Biol (2020) 30(6):478–90. doi: 10.1016/j.tcb.2020.02.009 PMC7230071 32413317

[24] Lei G Zhang Y Koppula P Liu X Zhang J Lin SH. The role of ferroptosis in ionizing radiation-induced cell death and tumor suppression. Cell Res (2020) 30(2):146–62. doi: 10.1038/s41422-019-0263-3 PMC7015061 31949285

[25] Kagan VE Mao G Qu F Angeli JP Doll S Croix CS. Oxidized arachidonic and adrenic PEs navigate cells to ferroptosis. Nat Chem Biol (2017) 13(1):81–90. doi: 10.1038/nchembio.2238 27842066 PMC5506843

[26] Seibt TM Proneth B Conrad M. Role of GPX4 in ferroptosis and its pharmacological implication. Free Radic Biol Med (2019) 133:144–52. doi: 10.1016/j.freeradbiomed.2018.09.014 30219704

[27] Tang D Chen X Kang R Kroemer G. Ferroptosis: molecular mechanisms and health implications. Cell Res (2020). doi: 10.1038/s41422-020-00441-1 PMC8026611 33268902

[28] Gao M Yi J Zhu J Minikes AM Monian P Thompson CB. Role of mitochondria in ferroptosis. Mol Cell (2019) 73(2):354–363 e353. doi: 10.1016/j.molcel.2018.10.042 30581146 PMC6338496

[29] Chen P Li X Zhang R Liu S Xiang Y Zhang M. Combinative treatment of β-elemene and cetuximab is sensitive to KRAS mutant colorectal cancer cells by inducing ferroptosis and inhibiting epithelial-mesenchymal transformation. (2020) 10(11):5107–19. doi: 10.7150/thno.44705 PMC7163451 32308771

[30] Zhang L Liu W Liu F Wang Q Song M Yu Q. Corrigendum to "IMCA induces ferroptosis mediated by SLC7A11 through the AMPK/mTOR pathway in colorectal cancer". (2020) 2020:6901472. doi: 10.1155/2020/6901472 PMC7641259 33194006

[31] Ursini F Maiorino M. Lipid peroxidation and ferroptosis: the role of GSH and GPx4. Free Radic Biol Med (2020) 152:175–85. doi: 10.1016/j.freeradbiomed.2020.02.027 32165281

[32] Weigand I Schreiner J Röhrig F Sun N Landwehr LS Urlaub H. Active steroid hormone synthesis renders adrenocortical cells highly susceptible to type II ferroptosis induction. Cell Death Dis. (2020) 11(3):192. doi: 10.1038/s41419-020-2385-4 32184394 10.1038/s41419-020-2385-4 PMC7078189

[33] Basudhar D Bharadwaj G Somasundaram V Cheng RYS Ridnour LA Fujita M. Understanding the tumour micro-environment communication network from an NOS2/COX2 perspective. Br J Pharmacol (2018) 176(2):155–76. doi: 10.1111/bph.14488 10.1111/bph.14488 PMC6295414 30152521

[34] Fujii J Osaki T. Involvement of nitric oxide in protecting against radical species and autoregulation of M1-polarized macrophages through metabolic remodeling. Molecules (2023) 28(2). doi: 10.3390/molecules28020814 PMC9861185 36677873

[35] Sun S Wu Y Maimaitijiang A Huang Q Chen Q. Ferroptotic cardiomyocyte-derived exosomes promote cardiac macrophage M1 polarization during myocardial infarction. PeerJ (2022) 10:e13717. doi: 10.7717/peerj.13717 35818358 PMC9270880

[36] Thomas DD Heinecke JL Ridnour LA Cheng RY Kesarwala AH Switzer CH. Signaling and stress: the redox landscape in NOS2 biology. Free Radic Biol Med (2015) 87:204–25. doi: 10.1016/j.freeradbiomed.2015.06.002 PMC4852151 26117324

[37] Wang R Geller DA Wink DA Cheng B Billiar TR. NO and hepatocellular cancer. Br J Pharmacol. (2020) 177(24):5459–66. doi: 10.1111/bph.14838 10.1111/bph.14838 PMC7707086 31423564

[38] Hofseth LJ Saito S Hussain SP Espey MG Miranda KM Araki Y. Nitric oxide-induced cellular stress and p53 activation in chronic inflammation. Proc Natl Acad Sci USA (2003) 100(1):143–8. doi: 10.1073/pnas.0237083100 PMC140909 12518062

[39] Dzavík V Cotter G Reynolds HR Alexander JH Ramanathan K Stebbins AL. Effect of nitric oxide synthase inhibition on haemodynamics and outcome of patients with persistent cardiogenic shock complicating acute myocardial infarction: a phase II dose-ranging study. Eur Heart J. (2007) 28(9):1109–16. doi: 10.1093/eurheartj/ehm075 17459901

[40] Hansel TT Kharitonov SA Donnelly LE Erin EM Currie MG Moore WM. A selective inhibitor of inducible nitric oxide synthase inhibits exhaled breath nitric oxide in healthy volunteers and asthmatics. FASEB J. (2003) 17(10):1298–300. doi: 10.1096/fj.02-0633fje 10.1096/fj.02-0633fje 12738811

[41] Hellio le Graverand MP Clemmer RS Redifer P Brunell RM Hayes CW Brandt KD. A 2-year randomised, double-blind, placebo-controlled, multicentre study of oral selective iNOS inhibitor, cindunistat (SD-6010), in patients with symptomatic osteoarthritis of the knee. Ann Rheum Dis. (2013) 72(2):187–95. doi: 10.1136/annrheumdis-2012-202239 23144445

[42] Basudhar D Glynn SA Greer M Somasundaram V No JH Scheiblin DA. Coexpression of NOS2 and COX2 accelerates tumor growth and reduces survival in estrogen receptor-negative breast cancer. Proc Natl Acad Sci USA (2017) 114(49):13030–5. doi: 10.1073/pnas.1709119114 PMC5724261 29087320

[43] Glynn SA Boersma BJ Dorsey TH Yi M Yfantis H Ridnour L. Increased NOS2 predicts poor survival in estrogen receptor-negative breast cancer patients. J Clin Invest. (2010) 120(11):3843–54. doi: 10.1172/JCI42059 PMC2964971 20978357

[44] Wei X Zhang B Liang X Liu C Xia T Xie Y. Higenamine alleviates allergic rhinitis by activating AKT1 and suppressing the EGFR/JAK2/c-JUN signaling. Phytomedicine (2021) 86:153565. doi: 10.1016/j.phymed.2021.153565 33945919

