# Supplementary figures and images for: Development of a prognostic model based on ferroptosis-related genes for colorectal cancer patients and exploration of the biological functions of NOS2 in vivo and in vitro
Source: Front Oncol. 2023 Jun 6;13:1133946. doi: 10.3389/fonc.2023.1133946 (PMC10280989; doi:10.3389/fonc.2023.1133946)

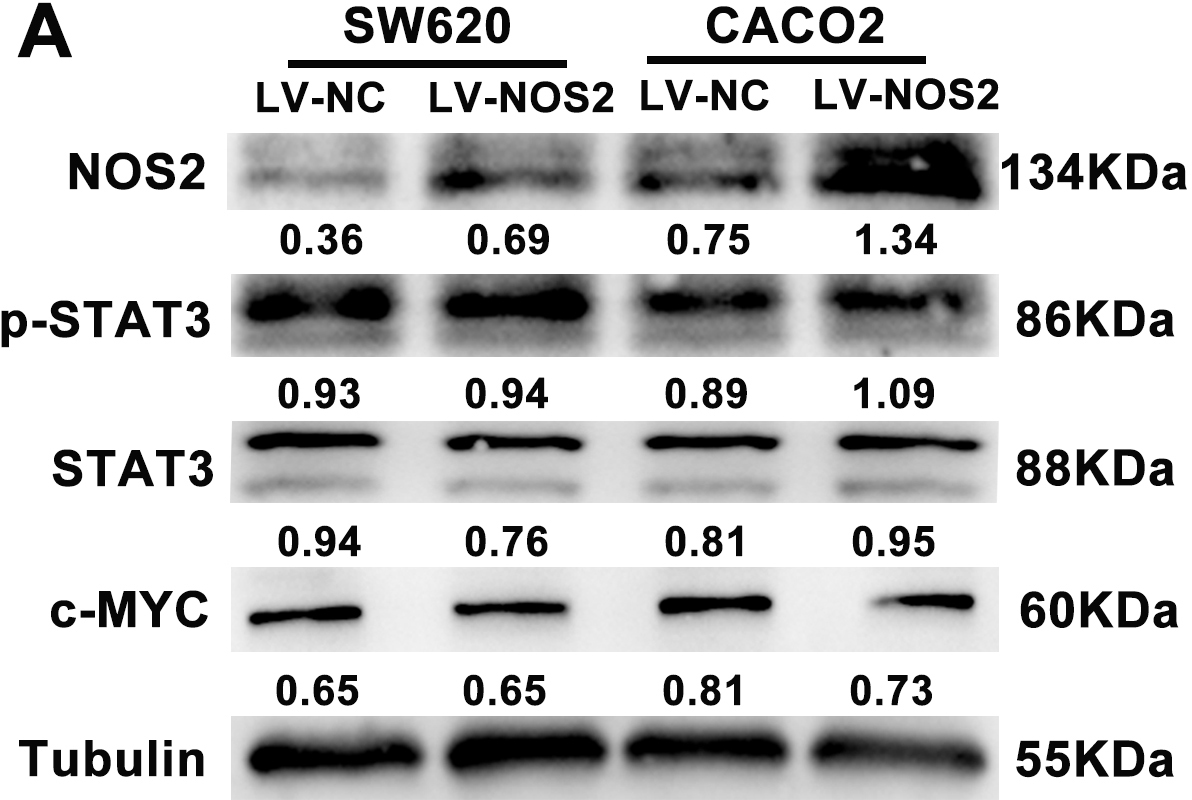

Supplement: Supplementary Figure 1 — Different NOS2 expression level hardly affected STAT3 and c-MYC signaling pathway. [file Image_1.jpeg]

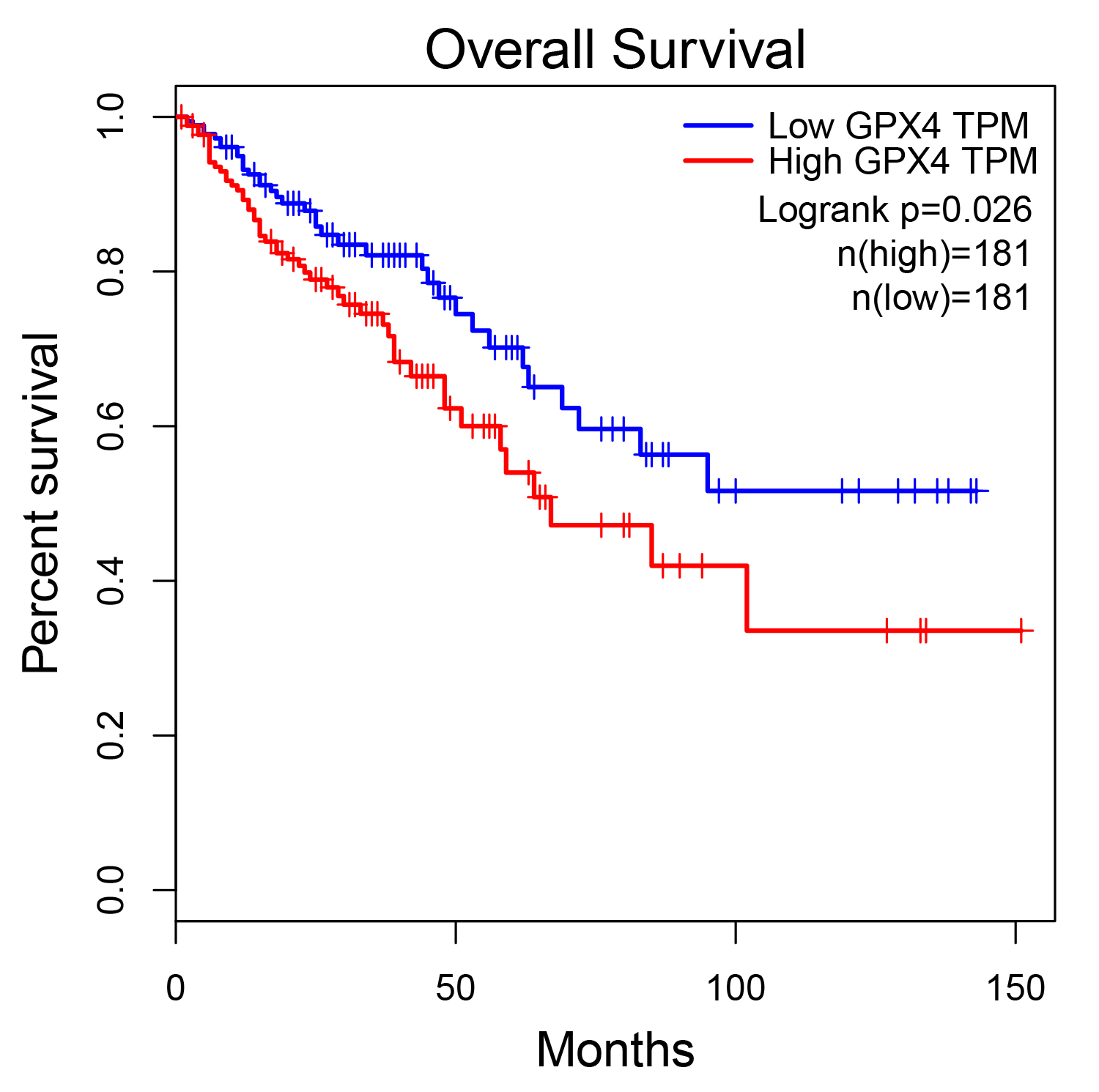

Supplement: Supplementary Figure 2 — Kaplan-Meier survival curves of OS with GPX4 expression level. [file Image_2.jpeg]
