# Supplementary material for: Development of a prognostic model based on ferroptosis-related genes for colorectal cancer patients and exploration of the biological functions of NOS2 in vivo and in vitro
Source: Front Oncol. 2023 Jun 6;13:1133946. doi: 10.3389/fonc.2023.1133946 (PMC10280989; doi:10.3389/fonc.2023.1133946)
Supplement: Supplementary file 3 [file Table_1.docx]

Supplementary Table 1. Identification of independent 8 FRGs by multivariate Cox regression analysis.

| Gene symbol | Hazard ratio | 95%CI | *p-*Value |
| --- | --- | --- | --- |
| NOS2 | 0.866 | 0.743-1.008 | 0.001 |
| DRD4 | 2.060 | 1.264-3.359 | 0.004 |
| STAT3 | 0.570 | 0.333-0.978 | 0.041 |
| LINC00336 | 3.346 | 1.037-10.792 | 0.043 |
| SLC2A3 | 1.539 | 1.230-1.926 | 0.001 |
| JDP2 | 2.029 | 1.255-3.280 | 0.001 |
| DUOX1 | 2.160 | 1.383-3.374 | 0.001 |
| ISCU | 2.706 | 1.470-4.982 | 0.001 |
